# Exploring effective patient feedback methods for eHealth in general practice

**DOI:** 10.1186/s12875-025-02725-0

**Published:** 2025-02-13

**Authors:** Mana Nasori, Marianne Mak-van der Vossen, Marije Holtrop, Jettie Bont

**Affiliations:** 1 Amsterdam UMC location AMC, Department of General Practice, Amsterdam Public Health, Quality of Care, and Personalized Medicine, Meibergdreef 9, Amsterdam, the Netherlands; 2 General Practitioners Holtrop and Westermann, Regional Organisation general practitioners Amsterdam (ROHA), Amsterdam, the Netherlands

**Keywords:** Patient feedback, General practice, eHealth, Feedback methods

## Abstract

**Background:**

The use of patient feedback is essential for identifying areas for improvement and tailoring care to the needs of patients, particularly in the context of eHealth, which has increased in adoption due to the pandemic. However, challenges persist in collecting feedback from vulnerable groups, those with severe conditions, or language barriers. Furthermore, concerns exist about the credibility and validity of the feedback received. This study aims to explore various possible forms that general practitioners (GPs) could use to collect patient feedback on eHealth applications in their daily practice.

**Methods:**

A Participatory Research (PR) was conducted involving an advisory group, patients, GPs and medical receptionists. The advisory group consisting of GPs, a board member, patient representatives and digital care manager affiliated with the primary care organisation ‘Regionale Organisatie Huisartsen Amsterdam’ (ROHA). The group provided input throughout the research process from the setup, data collection and interpretation to the finalization phase. Semi-structured interviews were conducted with 13 patients, 8 GPs and 2 medical receptionists. Participants were recruited through purposive sampling. Interviews were coded using Thematic Analysis.

**Results:**

Four themes were considered important. First, *timing of feedback*. Gathering instant feedback immediately after digital interactions was considered important. Secondly, the *feedback procedure*, whereby feedback should be given through the same communication channel as was used for the doctor-patient contact was valued. Also, participants preferred short and specific surveys, in which they can remain anonymous. Thirdly, for the *feedback content* some key feedback topics included general experiences, quality of care and technical aspects. The last theme was *advertisement*. Overall, patients do not want to burden their GP and thus tend to only give feedback if initiated by their GP. GPs themselves pointed out to have limited time for collecting feedback from patients due to their workload.

**Conclusion:**

GPs can optimize the feedback collection process by selecting targeted questions and integrating them into existing eHealth applications, thereby investing minimal time from GPs and patients. It is recommended to include automatic selected questions at the end of e-consultations. This integrated approach allows efficient feedback collection without burdening GPs.

**Supplementary Information:**

The online version contains supplementary material available at 10.1186/s12875-025-02725-0.

## Background

The use of patient feedback in healthcare can be challenging [1], but may potentially be valuable for improving quality of care [2]. One topic where patient feedback is of great importance is the continuously growing use of eHealth applications. However, there is little knowledge about the appropriate feedback method for different patient groups using eHealth applications in primary care. If we could identify which feedback method is best suited for eHealth and for which patient group, then general practitioners (GPs) could use patient feedback more effectively to improve their care. Gathering patient feedback on eHealth applications could provide valuable information to promote patient-centred care (PCC) and enhance general practice quality.

The active use of eHealth is believed to facilitate access to healthcare services and potentially enhance service efficiency. While the use of eHealth had already gradually increased in Dutch general practice [3], the pandemic has further intensified its adoption [4, 5] and it is now an essential part of care. Most GPs continue to use eHealth applications to provide care remotely [4, 5]. In this article we consider the following eHealth applications: requesting repeat prescriptions online, making an online appointment, accessing online test results, e-consultation and video consultation [3, 5]. Some of these applications have become indispensable in general practice and have affected patient care where care can be provided remotely. The GP is responsible for the quality of care that is delivered using these eHealth tools [6].

Overall GPs periodically collect patients’ experiences with healthcare and are often involved in quality improvement initiatives, such as quality circles. There are various forms for GPs to collect feedback from patients such as through, surveys, patient council, groups discussion (or focus groups) or waiting room interview, idea box or wish cards [1, 7]. Patient feedback is a valuable tool for GPs to identify areas for improvement and tailor care to meet the needs of patients. This is in line with PCC, defined as being responsive to patients’ values, preferences and their needs [8]. Surveys are the most commonly used methods to measure patients’ experiences [9, 10]. Although these surveys provide insight into what patients value most in their care and what areas need improvement, to our knowledge, it is unclear whether a survey is an appropriate method for measuring experiences with eHealth. Certain patient groups are unable to fill out surveys, including those who presumably face challenges with using eHealth applications. Therefore, there is a need to investigate which method would be appropriate.

From previous research, we know that using patient feedback effectively comes with numerous challenges. Research has identified concerns about asking vulnerable patients for feedback [11], appropriateness of feedback from patients who rarely visit their caregiver [12], difficulty in obtaining feedback from patients with severe conditions, from patients with language barriers [13], and issues with credibility and the validity of feedback [14]. Moreover, physicians may prefer real-time feedback and an overview of the average, but patients tend to provide either very positive or very negative feedback, making the average difficult to interpret and learn from [15]. Specific guidance is needed to facilitate reflection and discussion and foster learning [16], but aggregated opinions from patient groups limit the feedback’s specificity. These challenges also apply for collecting feedback about eHealth applications.

Previous studies have identified and prioritized numerous eHealth usability evaluation methods that assess the functional aspects, effectiveness and efficiency of eHealth systems [17, 18]. Remote User Testing has been recommended as the fastest eHealth usability evaluation method by Sinabell and Ammenwerth [17]. An additional study provided an overview of existing evaluation approaches for investigating eHealth usability and effectiveness. However, both evaluation methods are primarily intended for developers, researchers and evaluators [18]. Therefore, they may not be suitable for GPs to use in their daily practice. A recent report on the usability and effectiveness of digital healthcare applications in Dutch general practice care has been published [19]. While this study focused on which digital healthcare application works for which conditions and which patient characteristics [19], it does not give us answers to how patients’ experiences with eHealth could be collected. Furthermore, it more focused on the perspective of GPs than of patients [19]. As a result, it is still unclear how GPs can effectively collect patients’ experiences with eHealth applications in their daily practice, and which method is most appropriate for which patients.

Thus, there is a need to identify the most effective and appropriate feedback method for collecting patient feedback on eHealth applications in general practice. Therefore, the objective of this research is to explore feedback methods that GPs can use in their daily practice to collect patient feedback on eHealth applications and determine which method is most appropriate for various patients.

## Method

### Study design

Using a constructivist paradigm, meaning that there is no single objective truth and that multiple perspectives are valued to construct meaning, this study used a Participatory Research (PR) approach to use and explore various inputs, ideas, and experiences of stakeholders. In PR stakeholders are co-researchers during all phases of the research. This study started by formulating the research aim and question with the stakeholders. We created an advisory group to closely collaborate with stakeholders throughout the study. The advisory group consisted of six members: two GPs, one board member, two patient representatives from the patient council, and one manager of digital care of a primary care organisation named ‘Regionale Organisatie Huisartsen Amsterdam’ (ROHA). We collaborated with the advisory group for setup, data collection, analyses, and project finalization, to gather input and ideas on research questions, participation recruitment, and study outcomes. To ensure research integrity, members of the advisory group did not participate in the interviews.

### Setting

The study involved participants who are associated with ROHA. This organisation includes more than 200 GPs who provide primary care to approximately 400,000 patients in the Amsterdam region [20].

The role of the GP is of great importance in the Dutch healthcare system. Besides, GPs embody the most prominent group in the Dutch physician workforce. Generally, patients cannot see a medical specialist without a referral from their GP, and GPs are thus functioning as gatekeepers in the health care system. Dutch citizens are obliged to have health care insurance that covers GP care.

The majority of the GPs in the Netherlands work in private practices and are self-employed. However, they can be part of a GP care group such as ROHA [21].

This study was conducted at two general practices, both affiliated with ROHA.

### Reflexivity

This research project has been initiated by a district team affiliated with ROHA, who requested a scientific exploratory study for patient feedback on eHealth. The research team included a junior researcher (MN), a senior researcher and medical educator (MM), a GP, head of the general practice department (JB) and a general practitioner and board member at ROHA (MH). They are all affiliated with a large academic GP research group. Some of the researchers (JB/MM/MH) have been working in the GP setting for many years and are therefore familiar with the GP setting. During the entire project we strived for a reflective approach by carefully considering all perspectives. In order to avoid biased interpretation of data, we held frequent gatherings with the advisory group and research team, keeping a reflective stance and discussing our positions and their implications [22].

### Participants and recruitment

Patients from the two participating GP practices were considered eligible, including patients who do not use eHealth applications, to explore their familiarity with such applications. Eligible participants have various roles in eHealth patient feedback collection: GPs, managing patient feedback and using eHealth to provide care; medical receptionists, who typically interact with patients directly over the phone and receive verbal feedback about eHealth applications; and patients themselves, who are asked to provide feedback on the applications.

From May to July 2023, patients, GPs and medical receptionists from various general practices were recruited purposefully for this study. GPs were also recruited through snowballing. Patients who visited the GP were informed about the study by their GP and invited to participate by the main researcher (MN). Once informed consent was given, semi-structured interviews were conducted at the GP practice immediately after their consultation with their GP. Some patients preferred another time and location, which was planned with the main researcher (MN).

Through snowball sampling, two GPs from the advisory group shared an invitation with their district teams affiliated with ROHA to participate in the study. Additionally, GPs were recruited through an online networking platform for GPs called ‘HAweb’, specifically targeting the eHealth interest group.

The medical receptionists were also recruited through snowballing, two GPs shared the invitation with their medical receptionists, and responded if interested in taking part in the study. The interviews with GPs and medical receptionist were done online using Microsoft Teams. Data were collected until sufficiency had been reached, meaning that the collected data were comprehensive enough to answer the research question [23]. Data sufficiency was determined by coming to consensus within the research team.

### Data collection

We used semi-structured interviews in this qualitative study. A topic list covering the perspectives of patients and GPs, important themes for eHealth applications, and feedback methods, was used for semi-structured interviews (Additional file 1). The topic list was prepared with the advisory group who provided input for relevant topics to include. Interviews were done by the main researcher (MN) who is trained in conducting qualitative interviews. All interviews were audio-recorded and transcribed verbatim. Thereafter, the transcripts were pseudonymised and recordings were deleted.

### Data analysis

We used Thematic Analyses with continuous comparison to direct our analysis [24, 25]. Patient interviews were separately analysed from GP and medical receptionist interviews before comparing. First, two researchers (MN and MM) familiarized themselves with the data and then read and categorized relevant text fragments by attaching keywords (‘codes’) to them. The researchers kept notes (memos) of their thought process to ensure consistency and traceability. They collaborated to resolve any disagreements and consulted a third researcher (JB) if needed. Next, they created a coding tree based on their discussions and continued analysing the data using this tree. They searched for and refined themes, and the resulting themes were discussed with the entire research team to reach consensus. The emerging themes were discussed with the advisory group who provided input for interpretation of the data. The coding was done using MAXQDA software (version 2022).

### Use of large language models (LLM)

The author(s) have used ChatGTP during the preparation of the manuscript to improve the readability of it. The manuscript was edited and reviewed by all authors after using the LLM service, and if needed adapted the content.

## Results

The advisory group gathered three times in total to provide input in three phases of the study: [1] *Refining research idea and goal*. During this phase the advisory group and research team shared their reason and interest of being part of this research project, shared what they thought was important to focus on during the research; [2] P*roviding input for interpreting during data collection and analysis*. The preliminary data were shared with the advisory group, and we all discussed the interpretation of the data, the representation of the participant sample and whether perspectives or topics were missing that are relevant to the research question at hand; and [3] *Contributing in the finalization of the study*. During the final phase the entire research project gathered to discuss the research in its entirety and what the next steps are to realize collecting patient feedback on eHealth.

In total, we conducted 13 interviews with patients, 8 with GPs and 2 with medical receptionists. Interviews lasted between 8 and 45 min. Ten patients used eHealth applications, while three had never used it or had no intention to use eHealth applications. All eight GPs used eHealth applications and had also prior experience in collecting feedback from patients. The medical receptionists also had experience in collecting feedback from patients.

We describe the participants’ perspectives regarding an appropriate method for providing and collecting patient feedback on eHealth applications. Based on the data, the researchers were able to construct the following themes:1) timing of feedback 2) feedback procedure 3) feedback content and 4) advertisement.

### Timing of feedback

#### Instant feedback

Although patients and GPs are willing to give and collect patient feedback, they are reluctant to invest significant time and effort into the feedback process. They favour the feedback to be obtained immediately after the point of (digital) contact. For instance, after an e-consultation, there should be a feedback option available to provide feedback instantly. As patients tend to forget about their experiences if the GP asks for feedback weeks or months after the contact.

**Patient**

“*For example*,* I’m going to fill out a very long questionnaire. What is it? Fifteen minutes or so. And writing it all down*,* I wouldn’t do that. Unless you’re already in touch with the doctor*,* like over email. Because for me*,* it feels like closing the barn door after the horse has bolted”.* (Patient_23840)

**GP**

*“Yes*,* and it also takes time for the doctor. Well*,* I’m not sure if it’s really easy. Then it should actually be standard in your e-consultation response or something. Yes*,* otherwise you would have to send out a separate email or something.”* (GP_14895).

#### Specific limited period for collecting patient feedback

Some GPs mentioned that feedback from patients should be collected within a limited time frame. The main reason is that they do not have the time to process and act on the collected patient feedback, due to their heavy workload. In addition, they think patient feedback should be manageable for them to actively act on the feedback and should therefore be collected within a specific time frame. Thereby making patient feedback something more interesting and vividly, instead of collecting feedback on a daily basis. To achieve this, GPs suggested to only turn on the feedback feature during a limited feedback collection period.

**GP**

“*Yes*,* that you just put it on the agenda for colleagues to be focused on that for a while. “Well*,* then we have that project*,* the online repeat prescription improvement project. Let’s do that for two months and see how it goes”. That works better than receiving feedback notes all year round*,* I think. Yes.”* (GP_37921).

#### Collection by someone else

Some GPs had previous experience using a measuring tool to collect quality indicators for quality improvement. Due to their positive experience with this tool and interpreting the feedback, they expressed a preference for an external organisation to coordinate the feedback collection and to report the outcomes back to the GPs. Other GPs mentioned that a practice manager could take on the role of collecting verbal feedback, given the time-consuming nature of task. In addition, medical receptionists mentioned that patients already give feedback verbally by phone or in-person, as that is something that they are used to do. Therefore, they belief that providing verbal feedback to medical receptionists should stay possible and providing feedback electronically should be an additional option, as not every patient is able or feels comfortable to give written feedback. The important patient feedback will, as usual, be reported to the GP by the medical receptionist, if that is something the GP should take up on.

**GP**

*“ Then you are dealing with a hectic and busy day*,* yes*,* you could make a note somewhere and maybe if you have a practice manager*,* you can put them on it to sort that [feedback collection] out then. We don’t have a practice manager*,* so we have to do it all ourselves.*” (GP_17273).

**Medical receptionist**

“*It’s nice to receive feedback from patients. And when it’s digital*,* it becomes a bit less personal […]. Of course*,* eHealth applications are the future*,* I think the beauty lies in having both options. Some may prefer expressing themselves in writing and providing digital feedback*,* while others find it comfortable to do it in a more personal manner.”* (Medical receptionist 45668).

### Feedback procedure

#### Appropriate to recent use

Patients were asked their preferred method of providing feedback regarding their experiences with eHealth applications. Several patients stated that their choice depended on the digital communication channel being used at that moment. For example, if they received care through e-consultation, they would prefer to give feedback using the same channel instead of filling out paper surveys. Therefore, depending on the specific eHealth application being utilized, feedback should be requested or collected through that same application.

**Patient**

“*So*,* if I did an electronic consultation or such*,* if I would do an email exchange with my doctor*,* I would by modes of*,* in the last mail that I would then send to the doctor I would say what I think of the provision of care.”* (Patient_23840).

*“You could very simply maybe with an email*,* which you also have when you have a hairdressing appointment or something: rate this experience.”* (Patient_45282).

**GP**

*“…there is also less to gain in this area. But it is also easier to research. Yes. How it can be researched*,* I think it is just at the point in time*,* when people request their repeat prescription and then they go into that…they usually do that digitally in a portal*,* of course*,* that there might be a pop-up: What did you think?”* (GP_18199).

#### Short and specific

Patients prefer short surveys with specific questions; otherwise, they do not know what type of feedback they should provide, which makes it effortful to express their experiences. To address this, a survey should include questions with scores and an option to leave written remarks for specific feedback that was not covered in the survey. In addition, patients believe there should be a choice to provide feedback either anonymously or not. When it comes to personal experiences with healthcare in general, they prefer to leave it non-anonymously. As feedback regarding the use of eHealth is considered a smaller part of overall care, anonymity is deemed acceptable.

**Patient**

“*No*,* I think open-ended questions might be too much work.”* (Patient_45282).

**GP**

“*But if you’re already going to do three questions after a regular e-consultation*,* I don’t know if people feel like doing that. But that’s how it works. You want to know…it depends on what you want to know.”* (GP_22424).

### Feedback content

Both patients and GPs were asked about the topics they wanted to provide and receive feedback on. We categorized the feedback topics derived from the data into three themes (1) general experiences, (2) experiences related to quality of care and (3) technical aspects.

#### General experiences

Most GPs wish to gather the overall experiences of patients using eHealth applications, such as the overall satisfaction with eHealth as an option. They would like to know whether patients are satisfied with the possibility to use eHealth applications if needed. Moreover, GPs are interested in general improvement, for example whether patients experience any troubles and what they specifically ran up against.

#### Experiences related to quality of care

Some of the topics participants mentioned overlapped, such as the quality of care. All GPs expressed interest in knowing whether patients were adequately helped using e-consultation and if any follow-up actions were required. Similarly, patients wanted to provide feedback on whether they felt their GP had helped them adequately. Another shared topic was the speed of help, which both GPs and patients wanted to address in their feedback.

#### Technical aspects

Additionally, GPs found topics like usability, technical issues, and missing features of the application relevant. However, most GPs felt that they lacked control over the technical aspects, as those responsibilities lay with the product developer or supplier. Consequently, their role is limited to forwarding this information to the supplier. Nevertheless, some GPs had access to the product or system and the ability to adjust themselves, making their feedback on technical matters valuable.

Regarding patients’ feedback, some mentioned the desire to provide input on features that were not functioning properly, such as making online appointments.

### Feedback advertisement

#### Initiated by the GP

Most patients indicated that they would not give feedback to their GP unless the GP specifically asked for it. This tendency arises from the belief that GPs already have a heavy workload and, therefore, patients do not want to burden them with feedback. Asking for and providing feedback requires time and effort. In addition, some patients feel that they lack the authority to provide feedback, as they turn to their GPs seeking for medical help and consider them to have the essential knowledge and expertise. However, patients are willing to provide feedback if they believe it could be beneficial to their GP, and if the GP asked for it. Another reason mentioned was that patients could not come up with specific feedback topics on eHealth applications but are willing to give feedback if their GP asks for it.

During the interviews this particular topic was not mentioned by GPs.

**Patient**

“*Because you think*,* who am I to give that back. Maybe others think it’s normal*,* I can just deal with this or… yeah*,* so I think the GP has the position to initiate that*,* I think.”* (Patient_45282).

“*Yes*,* but if my opinion is neutral then you won’t hear anything. Unless you ask for it. And whether I will give feedback about digital contact I had with her (the GP)*,* not so much actually. No*,* actually not. Yes*,* unless the doctor asks for it…*” (Patient_23840).

#### Being aware of various patient feedback options

When patients were asked about their thoughts on providing patient feedback, most of them expressed unawareness concerning the possibility and methods to give feedback to their GP, despite their willingness to give feedback. Therefore, they tend to only consider sharing either extremely positive or extremely negative feedback, as they belief these are to be worth mentioning. They would choose to communicate such feedback either in-person or through the medical receptionist, as these are the only paths they are familiar with. However, patients think that they would be more aware of patient feedback as a possibility, if their GP would ask for feedback more often.

**Patient**

*“Or indeed everyone who goes to the GP gets an email once in a while with the opportunity to give your feedback and then different options with how you could do that. So*,* for the people who don’t want to do that face-to-face that they could do that online. Or I can imagine GPs don’t have the time to get 200 people face-to-face feedback*,* but still*,* I think it’s a very important part. You can just give better care if someone feels comfortable.”* (Patient_45282).

Similarly, GPs think that not all feedback methods are suitable for every patient. Some patients prefer providing written feedback, whereas others are more comfortable providing feedback in a personal conversation with their GP. Consequently, some GPs believe that it is important to raise awareness among patients about the various feedback options available, allowing them to choose the method they feel most at ease with. For instance, options such as feedback in-person, providing feedback electronically, leaving a note, and emphasizing that giving feedback is complete voluntarily. GPs pointed out that could be done by periodically mentioning the various feedback options through emails.

**GP**

“*Well*,* a good context about that*,* I think*,* information about that. I think you might explain yourself as a healthcare provider. Yes*,* and that you also offer different ways to give the feedback. So that with one… Yes*,* so that someone can do it digitally*,* but maybe you can also do it in an interview or on a sign-in column*,* verbally or on a note in the letterbox. So that the patient can choose which way they want to give the feedback.”* (GP_14895).

“*Yes. Or maybe a combination of a paper and a digital questionnaire? I think it would be problematic to just go digital*,* because then you immediately create a kind of separation. You only ask people who already want to fill in digital questionnaire.”* (GP_18199).

## Discussion and conclusion

The aim of our study was to explore various possible forms that GPs could use to collect patient feedback on eHealth applications in their daily practice. This qualitative study explored perspectives of patients, GPs and medical receptionists regarding an appropriate patient feedback method on eHealth applications in daily practice. We found four key themes that are considered important when collecting patient feedback on eHealth applications: timing of feedback, feedback procedure, feedback content and feedback advertisement.

### Timing of feedback

First of all, the timing of the feedback. Feedback should be collected instantly after the point of care, given the fact that when too much time has passed after the point of care, it is more difficult for patients to recall their experiences of that moment with eHealth applications. This finding is in line with the current trend in healthcare where experiences of patients are collected for quality improvement at the point of care, which is called real-time feedback (RTF) [26, 27]. Real-time feedback is usually collected through electronic devices which allows patients to give prompt feedback after their health experiences [26, 27]. Moreover, Sheard et al. (2019) reported that physicians had concerns about the timeliness of the collected feedback and preferred RTF [15], which corroborates with our finding. However, we also found that patients prefer giving instant feedback as well.

Some GPs expressed concerns about receiving feedback continuously as they do not have the time to check the feedback during a busy workday. Therefore, they suggested to gather patient feedback during a limited period where these features are temporarily switched on, which makes it more feasible and interesting to collect feedback on eHealth experiences. Carter et al. (2016) noted that for a successful implementation of RTF it should fit the practice routine of GPs and patients should not be over asked and therefore the time frame should be considered carefully [26]. The fact that patients did not mention this topic can be explained by the fact that they are not necessarily responsible for collecting feedback and quality improvement.

Furthermore, previous studies also pointed out that RTF is an efficient method to gather feedback which is important as healthcare professionals do not have dedicated time to collect feedback, especially with the current workload [28]. Similarly, this study showed that GPs and patients intend not to spend a lot of time collecting and providing patient feedback, therefore making the RTF method more suitable. Although studies suggest that RTF is beneficial for the patient-physician interactions, to our knowledge, it is ambiguous whether RTF method is effective for patients and GPs who use eHealth application in the GP-setting.

### Feedback procedure

In order to collect instant feedback from patients, both GPs and patients suggested to gather feedback through the specific eHealth application that is being utilised. To achieve this, participants proposed to build these feedback features into the patient portal where the feedback questions are automatically pop up after using that eHealth application.

Questions need to be short and specific and match with what the GP wants to know. For instance, some GPs pointed out to only select a subset of feedback questions that they wish to receive feedback on. These findings are consistent with previous studies [16, 29]. Loomis & Montague (2021) showed that physicians preferred immediate and specific directed feedback [29]. Similar to our findings, Baines et al. (2018) found that feedback’s specificity and inclusion of narrative comments influence the impact on positive behaviour change [16]. Interestingly, in our study also patients give preference to short and specific questions, as they do not want to spend too much time and effort giving feedback.

### Feedback content

Our study shows that feedback topics that should be considered when collecting patient feedback are technical aspects of eHealth applications and general experiences. Topics that are related to the quality of care should be part of the feedback method when asking patients about their experiences. However, these topics are only relevant with eHealth applications that influence the improvement of quality of care, such as e-consultation and video-consultation. Although most patients intend to provide feedback on technical aspects and general experiences with eHealth applications, most GPs expressed that these topics are less interesting for them as they cannot influence it directly. GPs can forward any technical issues regarding the eHealth applications to the developer. Besides, it might not be up to the GP to solve any technical issues as eHealth developers have their own helpdesk that patients can reach out to who have the essential expertise. However, we may argue that technical issues could hamper healthcare accessibility for which GPs have the responsibility, and therefore ought to at least be aware of these problems and to what extent the technical issues affect healthcare accessibility.

### Feedback advertisement

To assure effective feedback and collect feedback from different patient perspectives, various feedback methods should be made available as not every patient may feel comfortable to give feedback digitally. Our participants, GPs, medical receptionist as well as patients, suggested to offer various feedback methods to collect experiences from the entire population, because not every patient may be able or comfortable to provide written feedback. Likewise, Sanders et al. (2020) reported that feedback should be collected both digitally and instantly, including the possibility to give feedback at a later time using other methods such as paper surveys. Also the option to provide verbal feedback for patients who are unable to so in writing [13]. In addition, previous research has reported that vulnerable, low literate and non-native speaking patients are confronted with challenges in filling out surveys, requiring alternative methods [12–14]. However, this differs from the findings presented in our study. Our findings did not suggest providing alternative methods for vulnerable patients but rather to consider possible feedback procedures for those who feel comfortable when expressing themselves in writing. As GPs may lack the time to gather verbal feedback to reach their entire patient population, it is suggested that an external party or organisation takes this responsibility.

### Strengths and limitations

In our study, we included a variety of perspectives from participants with different roles such as patients, GPs and medical receptionists as they are the ones who are involved with patient feedback. The qualitative approach allowed us to explore and understand the thoughts and ideas of all the participants. In addition, the participatory approach allowed us to gather input from relevant stakeholders to formulate the research question, design the study and interpret the collected data to ensure that the outcomes are useful. However, a limitation of this study was that we did not make a distinguish between patients with and without digital literacy. Another limitation of this study is that we did not include non-native speakers, the low-literate patients and family members. As a result, we do not know what preferences they would have when it comes to giving feedback and using eHealth and if it would require other feedback mechanism.

### Future research

In this study, we explored various possible forms that GPs could use to collect patient feedback on eHealth in daily practice. Future studies could focus on how to collect feedback on eHealth applications from family members. For many users of eHealth applications, it is not the patient themselves but their family members who are the primary users. Therefore, family members should also be considered on what patient feedback method would be appropriate for this group. Besides, future studies could also focus on involving stakeholders when developing eHealth applications to consider what integration of patient feedback would be appropriate.

### Conclusion

This study set out to explore various possible forms of patient feedback on eHealth applications in general practice. It emphasizes that patients prefer feedback mechanisms that smoothly integrate with the care provision process, and that feedback mechanisms should be aligned with the eHealth applications being used at the time of care delivery. This finding highlights the need for feedback processes to be efficient and integrated into the workflow of care delivery.

Furthermore, this study shows that for new development in care delivery, such as using eHealth applications, require new forms of feedback mechanisms that it appropriate to how care is delivered. Moreover, it also shows that there is no there is no one-size-fits-all approach for collecting feedback from patients in eHealth applications. Instead, various approaches are required simultaneously to gather feedback on eHealth applications.

## Electronic supplementary material

Below is the link to the electronic supplementary material.

Supplementary Material 1

## Data Availability

The data that support our findings are available upon request from the authors. Due to sensitivity reasons data from this qualitative study are not publicly available. All data are stored in a controlled located storage of the Amsterdam UMC.
