# Supplementary material for: Exploring effective patient feedback methods for eHealth in general practice
Source: BMC Prim Care. 2025 Feb 13;26:40. doi: 10.1186/s12875-025-02725-0 (PMC11823149; doi:10.1186/s12875-025-02725-0)
Supplement: Supplementary file 1 — Supplementary Material 1 [file 12875_2025_2725_MOESM1_ESM.docx]

**Interview topic list - patient**

**Experiences**

*Experiences with patient feedback*

1. When you think of patient feedback (i.e. giving feedback to your GP), what is the first thing that comes to mind? (*examples/experiences)*
   1. What is your experience with giving feedback to your GP?
      1. How did it go?
      2. How was that for you?
2. What is your experience with eHealth in GP care? (example/experiences)
   1. What eHealth applications do you use?
      1. *Such as:*
         1. *E-consult*
         2. *Video consultation*
         3. *Telemonitoring*
         4. *Making appointments online*
         5. *Online prescription request*
         6. *Patient portal*
         7. *Digital self-triage*
   2. What is your personal experience with these eHealth applications?
      1. What are specific motivators and barriers to using the eHealth applications?
         1. Motivation to use
         2. Readability
         3. Findability/accessibility of the eHealth applications
         4. Complexity/completeness of eHealth applications, support needed
         5. Time
         6. Confidence in applying the eHealth application
         7. Prior experience in using eHealth application
      2. What makes you (do/don’t) use these eHealth applications?
      3. What goes well?
      4. What doesn’t go well?
      5. Are there things you should do differently? (if so, example?)

**Feedback themes eHealth applications**

1. What experiences with eHealth applications would you like to share with your GP?
   1. Such as:
      1. Technical aspects/usability of eHealth applications
      2. Experiences related to care delivered/patient-centred care/quality of care when using eHealth application
   2. What makes you want to share that with the GP?
   3. Are there any experiences you would not want to share with your GP?
      1. If so, what makes you do not want to share that?

**Method**

1. In what ways would you like to share your experiences about eHealth applications with your GP?
   1. *Such as:*
      1. *Questionnaire*
      2. *Email*
      3. *Online via the website*
      4. *Verbal*
      5. *By phone*
      6. *Through a colleague, assistant*
      7. *Combination*
   2. What makes you want to give feedback to the GP in this way?
2. Are there ways you would not use to share your experience(s) with the GP?
   1. What makes you do not want to share your experience(s) with the GP this way?

**Interview topic list - GP**

**Experiences**

*Experiences with patient feedback*

1. When you think of patient feedback (i.e. receiving feedback from a patient), what is the first thing that comes to mind? (*examples/experiences)*
   1. What is your experience with receiving feedback from patients? (*examples/experiences)*
      1. How did it go?
      2. How was that for you?
2. What is your experience with eHealth in GP care? (example/experiences)
   1. What eHealth applications do you use?
      1. *Such as:*
         1. *E-consult*
         2. *Video consultation*
         3. *Telemonitoring*
         4. *Making appointments online*
         5. *Online prescription request*
         6. *Patient portal*
         7. *Digital self-triage*
   2. What is your personal experience with these eHealth applications?
      1. What are specific motivators and barriers to using the eHealth applications?
         1. Motivation to use
         2. Readability
         3. Findability/accessibility of the eHealth applications
         4. Complexity/completeness of eHealth applications, support needed
         5. Time
         6. Confidence in applying the eHealth application
         7. Prior experience in using eHealth application
      2. What makes you (do/don’t) use these eHealth applications?
      3. What goes well?
      4. What doesn’t go well?
      5. Are there things you would do differently? (if so, example?)

**Feedback themes eHealth applications**

1. What experiences with eHealth applications would you like to receive from your patients?
   1. Such as:
      1. Technical aspects/usability of eHealth applications
      2. Experiences related to care delivered/patient-centred care/quality of care when using eHealth application
   2. What makes you want to know that from the patients?
   3. Are there any experiences you would not want to know from the patients?
      1. If so, what makes you do not want to share that?

**Method**

1. In what ways would you like to receive experiences about eHealth applications from patients?
   1. *Such as:*
      1. *Questionnaire*
      2. *Email*
      3. *Online via the website*
      4. *Verbal*
      5. *By phone*
      6. *Through a colleague, assistant*
      7. *Combination*
   2. What makes you want to gather feedback from patients in this particular way?
2. Are there ways you would not use to collect experience(s) of patients?
   1. What makes you do not want to collect experience(s) of patients this way?
